# Dolphin CONTINUE: a multi-center randomized controlled trial to assess the effect of a nutritional intervention on brain development and long-term outcome in infants born before 30 weeks of gestation

**DOI:** 10.1186/s12887-024-04849-1

**Published:** 2024-06-07

**Authors:** E. Janson, P. C. M. P. Koolschijn, L. Schipper, T. D. Boerma, F. N. K. Wijnen, W. P. de Boode, C. H. P. van den Akker, R. G. Licht-van der Stap, D. H. G. M. Nuytemans, W. Onland, S. A. Obermann-Borst, J. Dudink, C. G. M. de Theije, M. J. N. L. Benders, N. E. van der Aa

**Affiliations:** 1 Department of Neonatology, Wilhelmina Children’s Hospital, University Medical Center Utrecht, Utrecht, The Netherlands; 2 University Medical Center Utrecht Brain Center, Utrecht, The Netherlands; 3 Danone Nutricia Research, Utrecht, The Netherlands; 4 Institute for Language Sciences, Utrecht University, Utrecht, The Netherlands; 5 Department of Neonatology, Radboud University Medical Center, Radboud Institute for Health Sciences, Amalia Children’s Hospital, Nijmegen, The Netherlands; 6 Department of Pediatrics and Neonatology, Emma Children’s Hospital, Amsterdam University Medical Center, Location University of Amsterdam, Amsterdam, The Netherlands; 7 Amsterdam Reproduction and Development, Research Institute, Amsterdam University Medical Center, Amsterdam, The Netherlands; 8 Department of Neonatology, Maxima Medical Center, Veldhoven, The Netherlands; 9 Neonatology Network Netherlands, Amsterdam, The Netherlands; 10 Care4Neo, Patient and Parent Organization, Rotterdam, The Netherlands; 11 Department for Developmental Origins of Disease, University Medical Center Utrecht Brain Center, Utrecht, The Netherlands

**Keywords:** Nutrition, Preterm infant, Docosahexaenoic acid, Brain development, MRI, White matter integrity, Neurodevelopment, Language

## Abstract

**Background:**

Preterm born infants are at risk for brain injury and subsequent developmental delay. Treatment options are limited, but optimizing postnatal nutrition may improve brain- and neurodevelopment in these infants. In pre-clinical animal models, combined supplementation of docosahexaenoic acid (DHA), choline, and uridine-5-monophosphate (UMP) have shown to support neuronal membrane formation. In two randomized controlled pilot trials, supplementation with the investigational product was associated with clinically meaningful improvements in cognitive, attention, and language scores. The present study aims to assess the effect of a similar nutritional intervention on brain development and subsequent neurodevelopmental outcome in infants born very and extremely preterm.

**Methods:**

This is a randomized, placebo-controlled, double-blinded, parallel-group, multi-center trial. A total of 130 infants, born at less than 30 weeks of gestation, will be randomized to receive a test or control product between term-equivalent age and 12 months corrected age (CA). The test product is a nutrient blend containing DHA, choline, and UMP amongst others. The control product contains only fractions of the active components. Both products are isocaloric powder supplements which can be added to milk and solid feeds. The primary outcome parameter is white matter integrity at three months CA, assessed using diffusion-tensor imaging (DTI) on MRI scanning. Secondary outcome parameters include volumetric brain development, cortical thickness, cortical folding, the metabolic and biochemical status of the brain, and product safety. Additionally, language, cognitive, motor, and behavioral development will be assessed at 12 and 24 months CA, using the Bayley Scales of Infant Development III and digital questionnaires (Dutch version of the Communicative Development Inventories (N-CDI), Ages and Stages Questionnaire 4 (ASQ-4), and Parent Report of Children’s Abilities – Revised (PARCA-R)).

**Discussion:**

The investigated nutritional intervention is hypothesized to promote brain development and subsequent neurodevelopmental outcome in preterm born infants who have an inherent risk of developmental delay. Moreover, this innovative study may give rise to new treatment possibilities and improvements in routine clinical care.

**Trial registration:**

WHO International Clinical Trials Registry: NL-OMON56181 (registration assigned October 28, 2021).

## Background

Infants born (very and extremely) preterm are at risk for brain injury as they are born during a critical period of brain development. This so-called ‘encephalopathy of prematurity’ is mainly characterized by white matter injury [1] and may result in long-term neurodevelopmental deficits [2]. The encephalopathy is aggravated by dysmaturational events (including disturbances in neuronal, axonal and myelin formation), leading to widespread disturbances in white matter, cortical, and thalamic development, as identified in magnetic resonance imaging (MRI) studies [3, 4].

The plasticity of the brain during the first two years of life allows environmental factors, including nutrition, to influence brain development [5]. In the past, nutritional intake was studied solely for the purpose of catch-up growth and improved nutritional status. However, over the past 20 years, nutrition has received increasing attention as a potential intervention to also support developmental processes in the brain [6] and subsequent neurodevelopmental outcomes [7] in preterm infants. For example, during fetal and early postnatal development, the brain demands high amounts of energy and specific nutrients, such as amino acids, fatty acids, iron, zinc, and choline. This is needed to support specific neurodevelopmental processes, including cell proliferation, differentiation and metabolism, [8].

Previous studies suggest positive influences of human milk intake, glutamine supplementation, and long-chain polyunsaturated fatty acids (LCPUFAs) supplementation on white matter development in preterm infants (see for a recent review [6]). Regarding long-term neurodevelopmental outcomes, reviews show that single nutrient or macronutrient supplementation (mostly until term-equivalent age or a few months after discharge) has little to no effect on neurodevelopmental outcomes in preterm infants [9–11], except for supplementation of LCPUFAs, which seem to support visual attention and cognitive development [12, 13]. Additionally, the study by Dabydeen et al. [14] showed a beneficial effect of increased protein and energy intake for 12 months in infants with perinatal brain injury on neurological outcomes.

Supplementing specific nutrients critical for brain development over a longer period of time may be the best way to support both brain development and long-term outcomes in preterm infants. Docosahexaenoic acid (DHA), a LCPUFA, accumulates in the brain from the third trimester of pregnancy throughout the first year of life. Sufficient intake is required [15–17], as DHA deficiency is associated with impaired brain development and reduced neurodevelopmental outcomes [18–21]. In pre-clinical models, DHA has shown to dampen neuroinflammation [22], boost neurogenesis [23, 24], and promote neurite outgrowth and synaptogenesis [25, 26]. When combining DHA with other nutrients that occur naturally in human milk, namely uridine monophosphate (UMP) and choline, a synergistic effect on phospholipid synthesis is observed [27–29]. Thereby, supplementation of these three nutrients stimulates the formation of neuronal membranes, synapses [30–33] and sphingomyelin [27, 31], and this has shown promising effects on neurological outcome in (pediatric) models for brain injury [34, 35].

Two clinical pilot studies in preterm and term born infants [36, 37], who were at risk for or had established brain injury, showed a positive outlook on neurodevelopment following a similar dietary supplement, containing DHA, UMP, and choline, combined with eicosapentaenoic acid (EPA), arachidonic acid (ARA), cytidine monophosphate, vitamin B12, zinc, and iodine. Supplementation from birth up to two years of age was associated with clinically meaningful improvements in cognition and language at the age of 2 years [36, 37], and in visual attention at the age of 5 years [38]. However, little is yet known about the underlying effect of the nutritional supplement, as neuroimaging was not performed in these pilot studies. A recent report suggests an infant formula containing sphingomyelin, LCPUFAs and other potentially neuroactive ingredients affects myelinization in healthy term infants, as assessed using MRI scans after three and six months of age [39]. Neuroimaging can thus provide important insight into the mechanisms which may underlie a nutritional intervention.

The primary objective of the Dolphin CONTINUE (Concept Of Nutrition To Improve NeUrodevelopment in Early Life) study is to evaluate the effect of a nutritional intervention, containing DHA, UMP, and choline amongst others, from term-equivalent age up to 12 months CA, on white matter integrity in infants born before 30 weeks of gestation. We hypothesize that infants receiving the investigational product will have improved white matter integrity compared to infants receiving the control product, assessed using diffusion tensor imaging (DTI) (a specific MRI sequence) at three months CA. The secondary objectives are to determine the effect of the investigational product on safety, other MRI parameters, and behavioral-, motor-, cognitive-, and language development at 12 and 24 months CA.

## Methods/design

### Design

This is a randomized, placebo-controlled, double-blinded, parallel-group, multi-center trial. The study is sponsored by the University Medical Center (UMC) Utrecht. Consortium partners include Utrecht University, Health Holland, and Nutricia Research. Recruitment takes place in four different Neonatal Intensive Care Units in The Netherlands (UMC Utrecht, Amsterdam UMC, Radboud UMC, and Maxima Medical Center Veldhoven). The study is conducted within the Neonatology Network Netherlands (N3) organization (www.neonatology.eu) and supported by patient and parent organization Care4Neo (www.care4neo.nl). Study approval for all centers was provided by the Medical Ethical Committee of the UMC Utrecht. The study is registered in the WHO International Clinical Trials Registry (NL-OMON56181).

**Fig. 1:**
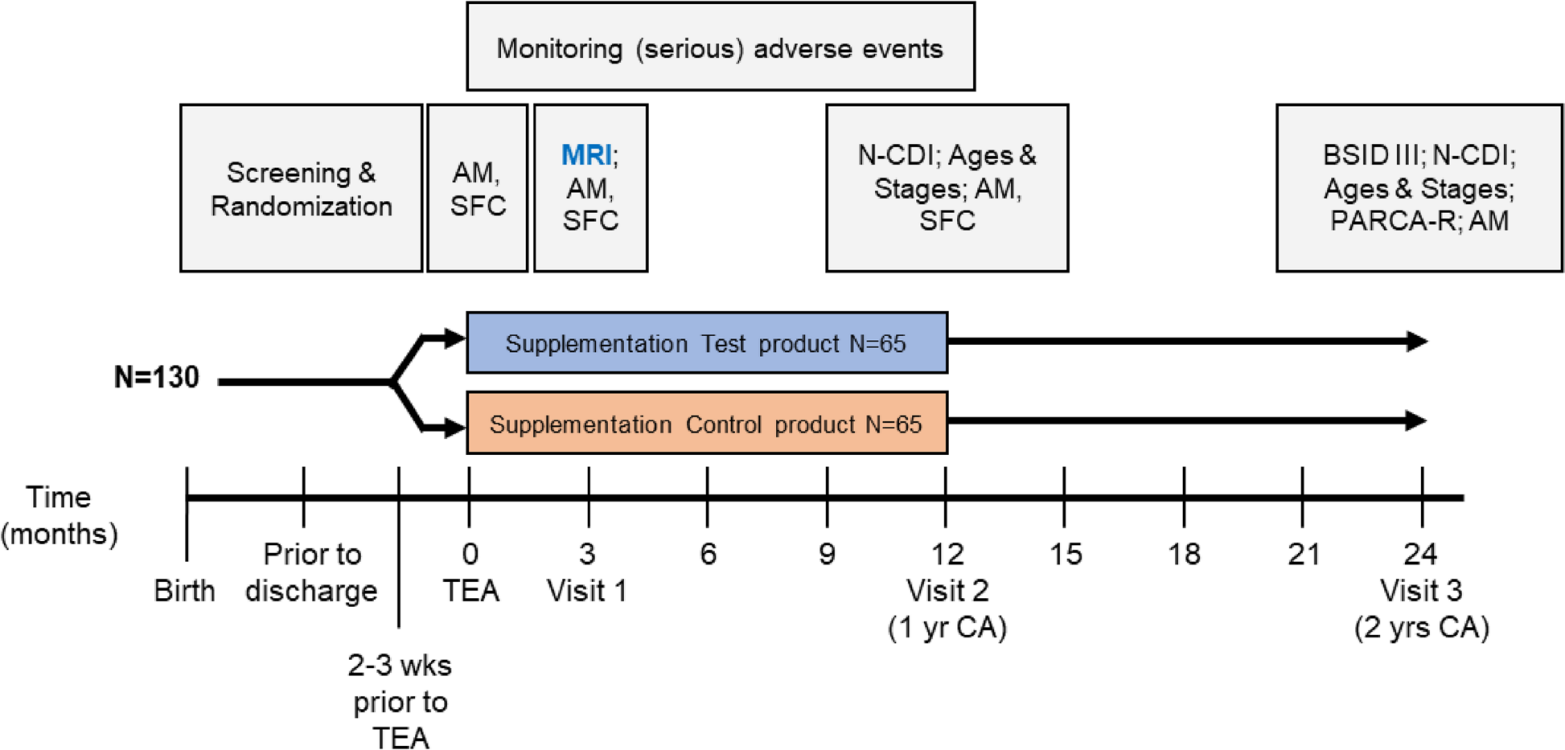
Study design and assessments per time point. AM = anthropometry, SFC = subject and feeding characteristics, MRI = magnetic resonance imaging, N-CDI = Dutch version of the Communicative Development Inventories, BSID-III = Bayley Scales of Infants Development III, PARCA-R = Parent Report of Child’s Abilities Revised, TEA = term-equivalent age, CA = corrected age

An overview of the study design and all study assessments is presented in Fig. 1. The nutritional intervention period will run from term-equivalent age (or at latest 3 weeks post-term) up to 12 months CA. Parents will provide the nutritional supplement themselves once the infant is at home after hospital discharge. If the infant is still admitted 3 weeks post-term, supplementation may start during hospital stay if parents are able and willing to provide the supplement themselves. At three months CA (Visit 1), a brain MRI scan will be made at the coordinating hospital (UMC Utrecht). At 12 and 24 months CA (Visit 2 and 3), growth and neurodevelopmental outcome will be assessed at the hospital of inclusion, as part of standard follow-up clinical care for preterm infants. In addition, digital questionnaires will be sent to caregivers to assess language, cognitive, motor, and behavioral development. During the intervention period, regular phone calls with caregivers will be held to obtain information on compliance, safety, anthropometry, subject and feeding characteristics.

### Study population

Infants with a gestational age at birth below 30 weeks are eligible for inclusion. An overview of the in- and exclusion criteria is provided in Table 1.

**Table 1:** Overview of the in- and exclusion criteria

**Inclusion criteria**
1. Preterm born infants born at a gestational age < 30 + 0 weeks 2. At least one parent masters the Dutch language 3. Written informed consent of caregivers
**Exclusion criteria**
1. Any relevant proven or suspected chromosomal anomaly, metabolic disorder or genetic syndrome 2. Presence of a congenital central nervous system infection or malformation (note that infants with *acquired* brain injury such as hemorrhages, white matter injury or stroke are eligible for inclusion) 3. Presence of any congenital gastrointestinal malformation (infants with a stoma following surgery are not necessarily excluded, at the discretion of the attending physician) 4. No realistic prospect of survival at the discretion of the attending physician 5. Expected or foreseen inability of the subject’s caregivers to adhere to protocol instructions 6. (Previous) participation in other nutritional intervention studies involving investigational or marketed nutritional products concomitantly or within three weeks prior to start study product intake, that could impact on the main outcome parameters and/or subject safety (at the discretion of the coordinating investigator) 7. Infants who are (suspected of) having a cow’s milk allergy and/or have already started with extensively hydrolyzed milk 8. Infants who are (suspected of being) allergic to eggs or fish oil (or products thereof) and/or lactose intolerant 9. Withdrawal of informed consent by caregivers 10. Infants who are not fully enterally fed and/or unlikely to start the nutritional intervention before 43 weeks’ postmenstrual age 11. Infants who are expected to be unable to undergo MRI under sedation at three months of CA

#### Treatment allocation and blinding

Randomization will be performed electronically in Castor EDC (Castor Electronic Data Capture, Amsterdam, The Netherlands, 2019; online available at: https://castoredc.com); the electronic case report form (eCRF)). The permuted block randomization will be stratified for sex and study site, using variable block sizes of 4 and 8. The allocation ratio will be 1:1 in investigational product group and control product group. Based on the order in which subjects enter the study within a site, and the stratification factors sex and site, the system will assign one of four product letter codes (A, B, C or D). Two random product codes (e.g., A and D) will correspond to the investigational product and two product codes (e.g., B and C) will correspond to the control product. In case of twins, the second infant will be allocated manually to the same product code as the first randomized sibling. Which codes correspond to which treatment is only known to the supplies manager at Danone Nutricia Research. The unblinding information will only be shared with the researchers performing the primary outcome analyses after pre-processing the statisticalanalysis of all MRI scans, and with those who need to treat the subject properly during a medical emergency that requires unblinding. All those involved in the implementation and/or decision making of the follow-up phase will remain blinded to study product allocation until the last infant completed the study.

### Study products

The active components in the investigational product are DHA, EPA, ARA, UMP, cytidine monophosphate (CMP), choline, zinc, vitamin B12 and iodine. The control product contains only fractions of the active components in the investigational product (Table 2). Both investigational and control products are isocaloric, have similar levels of fat and comparable energy content. The study products have been developed and will be produced by Danone Nutricia Research (Utrecht, the Netherlands) and manufactured according to good manufacturing practices as described in the FSSC 22000 standard.

**Table 2:** Amounts of the active ingredients in the test and control product (in 1 g powder). DHA = docosahexaenoic acid, EPA = eicosapentaenoic acid, ARA = arachidonic acid, UMP = uridine monophosphate, CMP = cytidine monophosphate

	**Investigational product**	**Control product**
**Macronutrient composition**		
Energy value (kcal)	5.3	5.1
Carbohydrates (mg)	572.4	607.0
Protein (mg)	89.2	88.0
Fat (mg)	289.7	256.0
Saturates (mg)	104.1	102.0
Monounsaturates (mg)	98.0	113.0
Polyunsaturates (mg)	87.6	41.0
**Active Nutrients**		
DHA (mg)	45.1	1.2
EPA (mg)	9.4	-
ARA (mg)	4.8	1.2
UMP (mg)	1.77	-
CMP (mg)	1.77	-
Choline (mg)	10.3	0.3
Zinc (µg)	502.3	11.5
Iodine (µg)	14.8	0.40
Vitamin B12 (ng)	118.2	5.4

### Supplementation

The study product is a powdered dietary supplement, which may be added to (expressed) human milk, standard preterm, infant or young child formula, and/or any normal foods. The recommended intake of test or control product is 1 g/kg body weight per day, with a maximum of 12 g per day. Dosage will be increased in a stepwise fashion per kilogram of bodyweight. It is recommended to divide the total amount of product per day across two feeds per day, for example a morning and an evening feed or what is most convenient for the caregiver and preferably at the same time each day.

### Outcome measures

#### Primary outcome

The primary outcome parameter in this study is white matter integrity at three months CA (i.e., after at least 12 weeks study intervention; Fig. 1). MRI scanning will be performed at the UMC Utrecht for all infants. Infants will be sedated using chloral hydrate to prevent motion artefacts. During examination and up to six hours after chloral hydrate administration, oxygen saturation, respiratory rate, and heart rate will be monitored. White matter integrity will be assessed using multi-shell DTI; an MRI technique which allows quantification of the degree of water diffusion parallel to the axons, referred to as fractional anisotropy (FA). Decreased FA values in preterm infants, scanned at term-equivalent age, have previously been associated with impaired motor and cognitive outcome at two years of age [40, 41]. Individual FA maps will be analyzed using tract-based spatial statistics (TBSS).

#### Secondary outcomes

Parental assessments of infant neurodevelopment using digital questionnaires (Fig. 1).

- Dutch version of the Communicative Development Inventories – Words and Gestures (N-CDI‐WG) [42] to assess development of early language, including vocabulary comprehension, production, gestures, and grammar, at 12 months CA;
- Dutch version of the CDI – Words and Sentences (N-CDI‐WS), at 24 months CA;
- Ages and Stages Questionnaire 4 (ASQ4), a currently being validated version of the ASQ3 [43], to assess communication, gross motor skills, fine motor skills, problem solving, and personal-social skills at 12 and 24 months CA;
- Parent Report of Children’s Abilities-Revised (PARCA-R; Dutch version [44]) to assess language and cognitive development at 24 months CA.

Physician assessments (Fig. 1):

- Bayley Scales of Infant and Toddler Development-III, Dutch version (BSID-III-NL) to clinically evaluate cognitive and fine and gross motor development at 24 months CA;
- Anthropometry (weight, length, and head circumference) at 3, 12, and 24 months CA.

Other MRI parameters at 3 months CA:

- TBSS on other DTI parameters (radial diffusivity, axial diffusivity, and mean diffusivity);
- Brain injury scores (white matter, cortical gray matter, deep gray matter, cerebellar, and global injury scores) will be assessed using the Kidokoro score [45];
- Brain tissue volumes (cerebellar, and (sub)cortical gray matter, unmyelinated white matter, deep nuclear gray matter, ventricular volumes, and cerebrospinal fluid) assessed using the automatic segmentation method by Makropoulos et al. [46];
- Cortical morphology (sulcation index and cortical thickness);
- Metabolic and biochemical status (quantification of several metabolites).

#### Safety outcomes

Occurrence, type, duration, and relationship with study product and seriousness of (serious) adverse events ((S)AEs) from start study product intake up to two weeks after the nutritional intervention period.

### Protocol compliance and missing data

Parameters for assessing protocol compliance will be checked during phone calls and visits, and entered in the eCRF. These include filling in a supplemental intake diary one week prior to scheduled phone calls, and visits during the intervention period. Caregivers have the right to withdraw their child from the study at any time without consequences. The coordinating investigator (NEvdA) can also decide to withdraw a child from the study for medical reasons, if the child develops any allergy for cow’s milk, egg (products) or fish oil (products), or if there are doubts regarding the ability of caregivers to comply to the protocol requirements. All data collected until subject discontinuation will be used. No new data will be collected after the moment of subject discontinuation, except for follow-up of safety data up to two weeks following subject discontinuation.

#### Data collection

Data will be collected and stored in conformance with EU data privacy laws, and details on data management are documented in the data management plan. All data will be stored in the eCRF. The data will be coded using unique subject codes which will not be retraceable to the individual infants. The site’s principal investigator will have the decoding key for infants included in their own center. The coordinating investigator will also have the decoding key for infants included in all centers.

MRI data will be stored in the Picture Archiving and Communication System (PACS) of the UMC Utrecht. Data of the electronic questionnaires – N-CDI and ASQ – will be stored on a secured network of Utrecht University, using the unique subject codes and with restricted access.

### Safety reporting

During the intervention period and up to two weeks thereafter, all (serious) adverse events ((S)AEs) for which a physician was involved or for which medication was given will be recorded. Deviations of more than 1 standard deviation from the child’s own, gestational age adjusted weight, length, or head circumference growth curve will also be recorded as an AE. All (S)Aes will be recorded in the eCRF. For safety monitoring, all SAEs will be reported, blinded, to the accredited Medical Ethical Committee and Danone Nutricia Research. The coordinating Investigator will submit, once a year throughout the clinical trial, a safety report to the accredited Medical Ethical Committee.

An independent Data Safety Monitoring Board (DSMB) will perform safety surveillance and check the assumptions of the sample size calculation to protect the scientific validity and credibility of the study and to advise upon adaptation of the sample size (if applicable).

Two interim analyses are planned (a first when 20 subjects will have completed MRI, and a second when 40 subjects will have completed the nutritional intervention) to evaluate safety outcomes, individual growth curves, and to check the assumptions on sample size estimation. All available safety data of all randomized subjects will be used for safety evaluation.

### Sample size calculation

Sample size is calculated based on previous simulations of the TBSS sensitivity for detecting treatment effects, as reported by Ball et al. [47]. When running TBSS on 45 subjects per group, TBSS detected 100% of the changes when the FA was increased with 20% and approximately 55% of the changes if the FA was increased with 5%. As we hypothesized an increase in FA by 5% following the intervention, we need at least 45 evaluable subjects per group.

However, not all infants are expected to continue supplementation until MRI scanning at three months CA. In previous studies, the drop-out rate was approximately 15% after 12 months of supplementation [36, 37]. In addition, despite sedation, we expect approximately 10% of MRI’s will (partially) fail due to motion artefacts. Due to the relatively high prevalence of twins/multiple births in the preterm population (approximately 20%), an inflation factor of 110% is applied to allow for clustering, as twins/multiple births will be randomized to the same treatment. In combination with the use of two stratification factors (following [48]), the total target population will consist of 130 randomized subjects.

### Statistical analyses

#### Primary outcome

Only infants who have a good quality DTI scan at three months CA and who have received at least 70% of the required study product prior to the MRI will be included in the primary analysis. The following hypothesis will be tested to assess the primary outcome:

- *H0: The effect of administering the investigational product is equal to the effect of administering the control product with respect to white matter microstructure integrity at 3 months of CA in preterm infants born* <*30*+*0 weeks of gestation.*
- *H1: The effect of administering the investigational product is unequal to the effect of administering the control product with respect to white matter microstructure integrity at 3 months of CA in preterm infants born* <*30*+*0 weeks of gestation.*

The hypothesis will be tested using TBSS [49] as implemented in FMRIB’s Software Library (FSL) (www.fsl.fmrib.ox.ac.uk/fsl). First, a population-specific tensor template will be created and iteratively optimized using the DTI Tool Kit (DTI-TK) [50, 51]. All individual tensor data will be registered to this template using rigid, affine, and diffeomorphic registration. After registration, individual tensor data will be transformed to individual FA maps in order to create a mean FA skeleton representing the center of all white matter tracts common to the group. This skeleton will be thresholded at FA > 0.15. Voxel-wise cross-subject statistical analysis will be performed using ‘Randomise’, which is part of the FMRIB’s Software Library. For the primary analysis, a general linear model will be used to assess the relationship between the FA and the intervention group (test vs control), whilst including the stratification factors (sex and study site), birth-weight *Z*-score, gestational age at birth, and postmenstrual age at time of scan as covariates. Family-wise error correction for multiple comparisons will be performed following threshold-free cluster enhancement. Only voxels with a *p*-value < 0.05 after these corrections will be regarded significant. Deblinding takes place after having performed the statistical analyses.

For sensitivity analyses, relevant clinical characteristics or confounders that differ significantly between the test and control group will be considered as additional covariates. The covariates might also be tested for their intervention modifying effect. In case the database reveals outliers, as identified by independent experts and/or statistical outlier identification, an additional analysis will be performed excluding these outliers. Reasons for outlier classification and results of these additional analyses will be described separately in the study report.

#### Secondary outcomes

The effects of the study product on the secondary outcome parameters will be studied using Student’s unpaired two-sample t-test or Chi-square for the test vs control product. Next, these effects will be further studied using linear regression analyses, introducing relevant clinical variables (e.g., gestational age at birth) as co-variates. For non-continuous outcome measures, (ordinary) logistic regression will be used. For longitudinal outcome measures, linear mixed effects modelling will be used. Outcome parameters with a discrete distribution will be analyzed with (stratified) non-parametric tests, generalized linear (mixed) models and/or Contingency Table Analysis. If needed, methods that can effectively handle censoring (survival analysis approaches) will be used. Investigation on possible confounder(s) and/or effect modifier(s) might be done. Before drawing conclusions, appropriate diagnostic tests will be conducted. In case of non-normally distributed data, transformations or non-parametric tests may be used. Safety parameters will be analyzed on the All Subjects Treated population. Further details on statistical analyses will be described in the statistical analysis plan (SAP) that will be finalized before database lock.

### Data sharing and communication of study results

Results will be submitted to peer-reviewed journals, independent of the results. If results are not accepted for publication, results will be published in another way (e.g., via international trial registers or open access data repositories). Once public interests have been secured, a plain language summary will be shared with caregivers of the subjects who participated in the study and to the general public via a neonatology patient association.

## Discussion

Encephalopathy of prematurity is common in extremely preterm born infants and associated with neurodevelopmental deficits. To date, no treatment options are available, but nutrition is a modifiable factor that may support brain development and thereby may improve long-term outcomes in preterm born infants. Supplementation of synergistic neurotrophic nutrients over a longer period of time may support white matter development and help overcome early brain damage. To our knowledge, this is the first randomized controlled trial to assess the influence of combined supplementation of DHA, UMP, and choline on both brain development, using MRI and neurodevelopmental outcome in preterm born infants.

We will use a relatively large sample size with a brain-derived primary outcome measure and multiple neurodevelopmental outcome assessments, including language, cognitive, motor, and behavioral development. Determining the effect of a nutritional intervention remains challenging as there are many other factors influencing brain and neurodevelopment, including differences in standard feeds, clinical factors [52, 53], and socio-economic status [54]. However, we try to control for these confounding factors by using an adequate sample size, performing randomization, and controlling for covariates in analyses.

In parallel, a large cohort study in the United Kingdom is currently assessing the effect of the same nutritional supplement on cognitive development in infants born before 28 weeks of gestation and in infants born after 35 weeks of gestation with hypoxic-ischemic encephalopathy (ISRCTN62323236, http://www.isrctn.com/ISRCTN62323236). Even though the inclusion criteria differ from our study and supplementation starts already at the neonatal unit, study results will be complementary. Combining study results will further elucidate the potential beneficial effect of synergistic nutrient supplementation in vulnerable infants at risk for, or with established brain injury.

If our study is successful, dietary supplementation will improve brain and cognitive development in preterm born infants. In addition, this study contributes to knowledge of the neurological underpinnings of neurocognitive development, thereby improving outcome predictions and adequate use of developmental interventions. Nutritional dosages of the study product are within the regulatory limits and product-related AEs were not observed in prior pilot studies. Hence, this study may ultimately give rise to a new and safe treatment possibility or change in routine clinical care to improve overall development in the vulnerable preterm population.

### Communication of trial results

Within one year after the end of the study, the study sponsor will submit a final study report including study results to the accredited Medical Ethical Committee of the UMC Utrecht. The study sponsor, together with two funding parties (Nutricia Research and Utrecht University), have agreed that the primary and secondary study results will be disclosed. Study results will be submitted to international peer-reviewed scientific journals, independent of whether study results will be positive or negative. Authorship will depend on individual contributions to the design, data collection, analyses of study results, and reviewing of the manuscript. If study results would unexpectedly not get accepted for publication, results will be published in another way, ensuring that the results are finable by others. This may include international trial registers or open access data repositories.

A plain language summary will be drafted and disseminated once publication interests have been secured, to share study results with study participants and with the general public via Care4Neo (the patient and parent organization).

## Data Availability

The datasets generated and/or analyzed during the current study will be available from the corresponding author on reasonable request, and will become available after publication in a yet to be determined repository.

## Declarations

Caregivers of eligible infants will be approached by one of the trained investigators and informed about the study prior to discharge on one of the participating neonatal intensive care units. Caregivers will obtain both verbal and written information about the study. After an appropriate amount of time for consideration and the possibility to ask questions, caregivers can make their own decision on whether or not to participate. Written Informed Consent will be obtained from the parents/guardian of the infants for the study. Caregivers can withdraw at any time of the study, without needing to provide a reason. In addition, infants will drop-out of the study if continued participation is a health risk for the infant.

## References

[1] Volpe JJ (2009). The encephalopathy of prematurity–brain injury and impaired brain development inextricably intertwined. Semin Pediatr Neurol.

[2] Kidokoro H (2014). Brain injury and altered brain growth in preterm infants: predictors and prognosis. Pediatrics.

[3] Volpe JJ (2019). Dysmaturation of premature brain: importance, cellular mechanisms, and potential interventions. Pediatr Neurol.

[4] Ortinau C, Neil J (2015). The neuroanatomy of prematurity: normal brain development and the impact of preterm birth. Clin Anat.

[5] Johnston MV (2009). Plasticity in the developing brain: Implications for rehabilitation. Dev Disabil Res Rev.

[6] Ottolini KM (2020). Nutrition and the developing brain: the road to optimizing early neurodevelopment: a systematic review. Pediatr Res.

[7] Schneider N, Garcia-Rodenas CL (2017). Early nutritional interventions for brain and cognitive development in preterm infants: a review of the literature. Nutrients.

[8] Georgieff MK, Brunette KE, Tran PV (2015). Early life nutrition and neural plasticity. Dev Psychopathol.

[9] Lin L (2019). Impact of macronutrient supplements for children born preterm or small for gestational age on developmental and metabolic outcomes: A systematic review and meta-analysis. PLoS Med.

[10] Hortensius LM (2019). Postnatal nutrition to improve brain development in the preterm infant: a systematic review from bench to bedside. Front Physiol.

[11] Chan SH (2016). Nutrition and neurodevelopmental outcomes in preterm infants: a systematic review. Acta Paediatr.

[12] Wang Q, Cui Q, Yan C (2016). The effect of supplementation of long-chain polyunsaturated fatty acids during lactation on neurodevelopmental outcomes of preterm infant from infancy to school age: a systematic review and meta-analysis. Pediatr Neurol.

[13] Fleith M, Clandinin MT (2005). Dietary PUFA for preterm and term infants: review of clinical studies. Crit Rev Food Sci Nutr.

[14] Dabydeen L (2008). High-energy and -protein diet increases brain and corticospinal tract growth in term and preterm infants after perinatal brain injury. Pediatrics.

[15] Farquharson J (1992). Infant cerebral cortex phospholipid fatty-acid composition and diet. Lancet (London, England).

[16] Makrides M (1994). Fatty acid composition of brain, retina, and erythrocytes in breast- and formula-fed infants. Am J Clin Nutr.

[17] Innis SM (2008). Dietary omega 3 fatty acids and the developing brain. Brain Res.

[18] Davis-Bruno K, Tassinari MS (2011). Essential fatty acid supplementation of DHA and ARA and effects on neurodevelopment across animal species: a review of the literature. Birth Defects Res B Dev Reprod Toxicol.

[19] Luchtman DW, Song C (2013). Cognitive enhancement by omega-3 fatty acids from child-hood to old age: findings from animal and clinical studies. Neuropharmacology.

[20] Neuringer M (1986). Biochemical and functional effects of prenatal and postnatal omega 3 fatty acid deficiency on retina and brain in rhesus monkeys. Proc Natl Acad Sci U S A.

[21] Champoux M (2002). Fatty acid formula supplementation and neuromotor development in rhesus monkey neonates. Pediatr Res.

[22] Sun GY (2018). Docosahexaenoic acid (DHA): An essential nutrient and a nutraceutical for brain health and diseases. Prostaglandins Leukot Essent Fatty Acids.

[23] Van Lo A (2019). Omega-3 docosahexaenoic acid is a mediator of fate-decision of adult neural stem cells. Int J Mol Sci.

[24] Sakayori N, Kimura R, Osumi N (2013). Impact of lipid nutrition on neural stem/progenitor cells. Stem Cells Int.

[25] Calderon F, Kim HY (2004). Docosahexaenoic acid promotes neurite growth in hippocampal neurons. J Neurochem.

[26] Cao D (2009). Docosahexaenoic acid promotes hippocampal neuronal development and synaptic function. J Neurochem.

[27] Cansev M (2009). Giving uridine and/or docosahexaenoic acid orally to rat dams during gestation and nursing increases synaptic elements in brains of weanling pups. Dev Neurosci.

[28] Wurtman RJ (2006). Synaptic proteins and phospholipids are increased in gerbil brain by administering uridine plus docosahexaenoic acid orally. Brain Res.

[29] Holguin S (2008). Chronic administration of DHA and UMP improves the impaired memory of environmentally impoverished rats. Behav Brain Res.

[30] Pooler AM (2005). Uridine enhances neurite outgrowth in nerve growth factor-differentiated PC12 [corrected]. Neuroscience.

[31] Cansev M (2008). Oral administration of circulating precursors for membrane phosphatides can promote the synthesis of new brain synapses. Alzheimers Dement.

[32] Cansev M (2015). Specific multi-nutrient enriched diet enhances hippocampal cholinergic transmission in aged rats. Neurobiol Aging.

[33] Savelkoul PJ (2012). A specific multi-nutrient formulation enhances M1 muscarinic acetylcholine receptor responses in vitro. J Neurochem.

[34] Brandt MJV (2021). Nutritional supplementation reduces lesion size and neuroinflammation in a sex-dependent manner in a mouse model of perinatal hypoxic-ischemic brain injury. Nutrients.

[35] Thau-Zuchman O (2019). Brain phospholipid precursors administered post-injury reduce tissue damage and improve neurological outcome in experimental traumatic brain injury. J Neurotrauma.

[36] Andrew MJ (2018). Neurodevelopmental outcome of nutritional intervention in newborn infants at risk of neurodevelopmental impairment: the Dolphin neonatal double-blind randomized controlled trial. Dev Med Child Neurol.

[37] Andrew MJ (2018). Nutritional intervention and neurodevelopmental outcome in infants with suspected cerebral palsy: the Dolphin infant double-blind randomized controlled trial. Dev Med Child Neurol.

[38] Atkinson J (2022). Visual attention and dietary supplementation in children with perinatal brain injury. Dev Med Child Neurol.

[39] Schneider N (2022). A nutrient formulation affects developmental myelination in term infants: a randomized clinical trial. Front Nutr.

[40] van Kooij BJ (2012). Neonatal tract-based spatial statistics findings and outcome in preterm infants. AJNR Am J Neuroradiol.

[41] Girault JB (2019). White matter microstructural development and cognitive ability in the first 2 years of life. Hum Brain Mapp.

[42] Zink M, Lejaegere M (2002). N-CDI's: lijsten voor communicatieve ontwikkeling. Aanpassing en hernormering van de MacArthur CDI's van Fenson et al.

[43] Squires J, Bricker D, Ages & Stages Questionnaires®, Third Edition (ASQ- 3™) (2009). A parent-completed child-monitoring system.

[44] Vanhaesebrouck S (2014). Cognitive assessment of very low birth weight infants using the Dutch version of the PARCA-R parent questionnaire. Early Hum Dev.

[45] Kidokoro H, Neil JJ, Inder TE (2013). New MR imaging assessment tool to define brain abnormalities in very preterm infants at term. AJNR Am J Neuroradiol.

[46] Makropoulos A (2018). The developing human connectome project: a minimal processing pipeline for neonatal cortical surface reconstruction. Neuroimage.

[47] Ball G (2013). Testing the sensitivity of tract-based spatial statistics to simulated treatment effects in preterm neonates. PLoS One.

[48] Silcocks P (2012). How many strata in an RCT? A flexible approach. Br J Cancer.

[49] Smith SM (2006). Tract-based spatial statistics: voxelwise analysis of multi-subject diffusion data. Neuroimage.

[50] Zhang H (2007). Unbiased white matter atlas construction using diffusion tensor images. Med Image Comput Comput Assist Interv.

[51] Zhang H (2007). High-dimensional spatial normalization of diffusion tensor images improves the detection of white matter differences: an example study using amyotrophic lateral sclerosis. IEEE Trans Med Imaging.

[52] Parikh NA (2021). Perinatal risk and protective factors in the development of diffuse white matter abnormality on term-equivalent age magnetic resonance imaging in infants born very preterm. J Pediatr.

[53] Barnett ML (2018). Exploring the multiple-hit hypothesis of preterm white matter damage using diffusion MRI. Neuroimage Clin.

[54] Benavente-Fernandez I, Siddiqi A, Miller SP (2020). Socioeconomic status and brain injury in children born preterm: modifying neurodevelopmental outcome. Pediatr Res.

